# Inhibition of CEBPB Attenuates Lupus Nephritis via Regulating Pim-1 Signaling

**DOI:** 10.1155/2022/2298865

**Published:** 2022-09-20

**Authors:** Xiaoyang Wang, Weili Cheng, Xiaopan Chen, Yanan Gong, Guangjie Wang, Xiaoxue Zhang, Yuanyuan Qi

**Affiliations:** ^1^Department of Nephrology, The First Affiliated Hospital of Zhengzhou University, 1#, Jianshe East Road, Zhengzhou, 450052 Henan, China; ^2^Department of Obstetrics and Gynecology, Third Affiliated Hospital of Henan University of Traditional Chinese Medicine, 63#, Dongming Road, Zhengzhou, 450003 Henan, China

## Abstract

Systemic lupus erythematosus (SLE) is an autoimmune disease leading to inflammatory damage in multiple target organs, and lupus nephritis (LN) is one of the most life-threatening organ manifestations. CCAAT/enhancer-binding protein *β* (CEBPB) regulates the NLRP3 inflammasome and is involved in the pathogenesis of SLE. However, the role and mechanism of CEBPB in LN remains unclear. MRL/lpr mice and lipopolysaccharides (LPS) combined with adenosine triphosphate- (ATP-) treated glomerular podocytes were used as models of LN *in vivo* and *in vitro*, respectively. *In vivo*, we investigated the expressions of CEBPB during the development of MRL/lpr mice. Then we assessed the effect of CEBPB inhibition on renal structure and function through injecting sh*CEBPB* lentivirus into MRL/lpr mice. *In vitro*, glomerular podocytes were treated with *Pim-1*-OE and si*CEBPB* to explore the relation between *CEBPB* and *Pim-1*. The progression of LN in mice was associated with the increased level of CEBPB, and the inhibition of CEBPB ameliorated renal structure impairments and improved renal function damage associated with LN. Knockdown of *CEBPB* could suppress the activation of NLRP3 inflammasome and the secretion of IL-1*β* and IL-6. Furthermore, the knockdown of *CEBPB* could inhibit NLRP3 inflammasome activation and pyroptosis via binding to *Pim-1* promoter to downregulate its expression, and the overexpression of *Pim-1* reversed the effects of *CEBPB* deficiency. The regulation of *CEBPB* on *Pim-1* facilitated pyroptosis by activating NLRP3 inflammasome, thereby promoting the development of LN.

## 1. Introduction

Systemic lupus erythematosus (SLE) is an autoimmune disease characterized by the production of numerous autoantibodies against nuclear antigens and immune complex deposition, leading to inflammatory damage in multiple target organs. One of the most life-threatening organ manifestations is lupus nephritis (LN), and it is the main cause of morbidity and mortality in SLE [1]. The etiology and pathogenesis of LN are multifactorially and incompletely understood. Available evidence indicates that tubulointerstitial injury, especially the damage of proximal tubule epithelial cells, contributes to the occurrence and development of LN [2]. The activation of inflammasome in both SLE patients and murine macrophages enhances the inflammation and autoimmunity, resulting in LN flaring and organ damage [3, 4]. Although the application of corticosteroids or other immunosuppressive drugs has improved the prognosis of LN patients, the use of these treatments are often accompanied by drug resistance and treatment-related severe adverse effects. Therefore, there is an urgent need to deeply explore the underlying pathogenesis of LN and search for novel targets for the therapy of LN.

CCAAT/enhancer-binding protein *β* (C/EBP*β*, CEBPB), a member of the CCAAT/enhancer-binding protein, acts as a b-ZIP transcription factor that participates in native immunities. According to reports, the increased expression of CEBPB in peripheral blood mononuclear cells of SLE patients is positively correlated with disease progression, suggesting that CEBPB may be involved in the pathogenesis of SLE, but its specific effects on LN remain unclear [5]. In addition, *NLRP3* has been reported to be an important gene that could activate inflammasomes. The activation of NLRP3 inflammasome is often accompanied by the activation of the cell death pathway in form of pyroptosis, apoptosis, and necroptosis, and it contributes to the occurrence of LN [6]. A recent study has shown that the expression of NLRP3 is suppressed in peritoneal macrophages from *CEBPB*^−/−^ mice, and *CEBPB* knockdown in macrophages can inhibit systemic inflammation [7].

The family of Pim serine/threonine kinase is composed of three highly conserved isoforms namely Pim-1, Pim-2, and Pim-3. They have special structural characteristics that encode serine/threonine kinases with a broad range of cellular targets. Pim kinases, as potent oncogenes, could uniquely activate toward cell proliferation, apoptosis, and the cancer-specific metabolism. Thus, Pim proteins become appealing targets in cancer and autoimmune disease [8]. Upregulation of Pim-1 is found in peripheral blood mononuclear cells of SLE patients and renal biopsy tissue of LN patients. Pim-1 modulates the activation of NLRP3 inflammasome via the regulation of intracellular Ca^2+^. Moreover, inhibition of Pim-1 suppressed the inflammasome activation in mouse and human glomerular podocytes and reduced LN symptoms [9]. However, the role of CEBPB in the regulation of Pim-1 in LN has not been reported.

In the present study, we identified that knockdown of *CEBPB* alleviated the development of LN, suppressed the activation of the inflammasome, and downregulated the expression of *Pim-1* in mouse and glomerular podocytes. These findings suggest that targeting CEBPB may be a helpful approach for the improvement of LN.

## 2. Materials and Methods

### 2.1. Lupus Models

The animal experiments were performed following the National Institutes of Health Guide for the Care and Use of Laboratory Animals (NIH Publications No. 8023, revised 1978). All procedures were approved by the Ethics Committee of the First Affiliated Hospital of Zhengzhou University. 24 MRL/MPJ (control; female) mice and 36 MRL/lpr (LN; female) mice were used in this study (6 mice for each group). MRL/MPJ and MRL/lpr mice at the age of 9 weeks were randomly maintained in the facility at 25 ± 1°C and 45-55% constant humidity with water and food available.

### 2.2. Treatment Protocols

After a week of adaptation, mice were sacrificed by intraperitoneal injection of 200 mg/kg sodium pentobarbital at 10th, 14th, and 18th week, and the samples of blood, urine, and kidney were collected for analysis. To detect the role of CEBPB in the development of LN, MRL/lpr mice were randomly divided into two groups. They were injected with 2 × 10^8^ TU of *CEBPB*-short hairpin RNA lentivirus (sh*CEBPB*-LV group) or negative control lentivirus (NC-LV group) from the tail intravenously at 10th and 14th week. Meanwhile, the same dose of phosphate-buffered solution (PBS) was processed on the MRL/MPJ mice served as the congenic controls (Control group). At the end of the 18th week after the treatment, the mice were sacrificed by 200 mg/kg sodium pentobarbital and the blood, urine, spleen, and kidney of the mice were collected.

### 2.3. Cell Culture

Conditionally immortalized mouse podocytes were purchased from iCell Bioscience Inc. (Shanghai, China), and were cultured in RPMI-1640 medium (cat. 31800, Solarbio, Beijing, China) with 10% fetal bovine serum (cat. 11011-8611, Tianhang, Zhejiang, China) and added recombinant mouse IFN-*γ* (20 U/mL, cat. P01580, Sino Biological Inc., Beijing, China). Before experiments, cells were removed IFN-*γ* and grown at 37°C for 14 days to induce phenotype differentiation. Two *CEBPB* interference fragments (si*CEBPB*-1, si*CEBPB*-2) were transfected into the differentiated glomerular podocytes by using liposome 3000 (cat. L3000-008, Invitrogen, Carlsbad, USA), the podocytes were primed with lipopolysaccharides (LPS, 200 ng/mL, cat. SA9730, Solarbio, Beijing, China) for 4 h after transfected for 48 h and subsequently stimulated with adenosine triphosphate (ATP, 5 mM, cat. L8880, Solarbio, Beijing, China) for 1 h. To explore the relationship between *CEBPB* and *Pim-1*, a *Pim-1* overexpression vector (*Pim-1*-OE) was constructed. The differentiated glomerular podocytes were cotransfected with *Pim-1*-OE and si*CEBPB* for 48 h, primed with LPS (200 ng/mL, cat. SA9730, Solarbio, Beijing, China) for 4 h and then stimulated with ATP (5 mM, cat. L8880, Solarbio, Beijing, China) for 1 h.

### 2.4. Renal Function Detection and Enzyme-Linked Immunosorbent Assay (ELISA)

The levels of 24-hour proteinuria (cat. C035, Jiancheng Bioengineering Institute, Nanjing, China), serum creatinine (cat. C011, Jiancheng Bioengineering Institute, Nanjing, China), and blood urea nitrogen (BUN) (cat. C013, Jiancheng Bioengineering Institute, Nanjing, China) were measured by detection kits according to the manufacturer's protocol.

The levels of serum anti-dsDNA (cat. CSB-E11194m, CUSABIO, Houston, USA) and complement C3 (cat. EM0319, Wuhan Fine Biotech Co., Ltd., Wuhan, China), as well as the level of IL-1*β* (cat. EK201B, Invitrogen, Carlsbad, USA) and IL-6 (cat. EK206, Invitrogen, Carlsbad, USA) in the cell supernatant were determined by ELISA kits. All the experiments were implemented under the manufacturer's instruction.

### 2.5. Histopathological Analysis

Histological changes of kidney tissues were detected by Hematoxylin-eosin (H&E) and Periodic Acid-Schiff (PAS) staining. The obtained kidneys were dehydrated in different concentrations of alcohol and vitrified in dimethyl benzene, and then embedded in paraffin blocks. The 5 *μ*m renal sections were stained with H&E and PAS, respectively. The microstructure changes were observed under a light microscope (BX53, Olupums, Tokyo, Japan). The presence of inflammatory cell infiltration observed by H&E staining was scored from zero to four (zero, absent; one, less than 25% of the section; two, 25-50% of the section; three, 50-75% of the section; four, more than 75% of the section) [10]. The severity of glomerular pathology correlated with the deposition of PAS-positive materials. PAS-positive deposits were graded from zero to four (zero, absent; 1, mild; 2, mild-moderate; 3, moderate; 4, severe) [10].

### 2.6. Immunofluorescence Staining

Frozen kidney samples were fixed with acetone and embedded in paraffin. The cryostat sections (5 *μ*m) were baked at 60°C for 2 h and washed with PBS for three times after being deparaffinized with xylene and rehydrated by graded alcohol. Subsequently, the sections were blocked for 15 min with goat serum at room temperature and then incubated with the primary antibody overnight at 4°C. The primary antibodies were as follows: IgG (1 : 100, cat. ab172730, Abcam, Cambridge, UK), NLRP3 (dilution 1 : 50, cat. NBP2-03948SS, Novus Biologicals, Littleton, USA) and CEBPB (dilution 1 : 50, cat. sc-7962, Santa Cruz, Shanghai, China). After being washed with PBS for three times, the slides were incubated with Cy3-labeled antibodies (goat antirat, cat. A0516, Beyotime, Shanghai, China) or FITC-labeled antibodies (goat antimouse, cat. A0562, Beyotime) at room temperature preventing from the light. After washing again, the slides were counterstained with 4,6-diamidino-2-phenylindole (DAPI) that was used to visualize the nuclei. Last, the images were observed and captured utilizing a fluorescence microscope (BX53, Olupums, Tokyo, Japan).

### 2.7. Reverse Transcription-Quantitative PCR (qRT-PCR)

Total RNA from cells or kidney tissue was extracted by the Trizol method according to the manufacturer's instructions and converted to cDNA using the Super M-MLV Reverse Transcriptase (cat. PR6502, BioTeke, Beijing, China). qRT-PCR was performed in triplicate using Taq PCR MasterMix (cat. PC1150, Solarbio, Beijing, China) in a reaction system of 20 *μ*L. A real-time PCR system (Exicycler 96, BIONEER, South Korea) was used to amplify and the relative gene expression changes were calculated according to the formula of 2^−∆∆ct^. The primers for the amplifications were listed as follows.

*CEBPB* forward: 5′-CCAAGAAGACGGTGGACAAGC-3′, reverse: 5′- GAACAAGTTCCGCAGGGTGC-3′ and *Pim-1* forward: 5′- TTCGGCTCGGTCTACTCT-3′ and reverse: 5′-TCCTCGTTCGGTGATAAAG-3′.

### 2.8. Western Blotting

Renal tissues and cells were lysed with RIPA lysis buffer (cat. P0013, Beyotime, Shanghai, China) and protein concentration was measured by BCA Protein Concentration Kit (cat. P0011, Beyotime, Shanghai, China). The same amount of protein was loaded to 5% sodium dodecyl sulfate-polyacrylamide gel separated for 2.5 h and then transferred to polyvinylidene difluoride membranes. After blocking for 1 h at room temperature, the membranes were incubated with primary antibodies at 4°C overnight. Secondary, the blots were washed and incubated with the corresponding IgG-HRP secondary antibodies. The signals on the membranes were visualized via the chemiluminescence kits (cat. P0018, Beyotime, Shanghai, China) and quantified by Gel-Pro-Analyzer (Media Cybernetics, USA). The antibodies were as follows: NLRP3 (dilution 1 : 1000, cat. A12694, ABclonal, Shanghai, China); p20 Caspase-1 (dilution 1 : 1000, cat. AF4005, Affbiotech, Cincinnati, OH, USA); p17 IL-1*β* (dilution 1 : 1000, cat. A16288, ABclonal, Shanghai, China); CEBPB (dilution 1 : 1000, cat. AF6202, Affbiotech, Cincinnati, USA); Cleaved Gasdermin D (dilution 1 : 1000, cat. 10137, CST, Danvers, USA); *β*-actin (dilution 1 : 1000, cat. sc-47778, Santa Cruz, Shanghai, China); Goat antirabbit IgG (dilution 1 : 5000, cat. A0208, Beyotime, Shanghai, China); Goat antimurine IgG (dilution 1 : 5000, A0216, Beyotime, Shanghai, China).

### 2.9. Chromatin Immunoprecipitation Assay

The chromatin immunoprecipitation (ChIP) assay was performed by using a ChIP assay kit (cat. WLA106a, Wanleibio, Shenyang, China) according to the manufacturer's instructions. Briefly, 37% formaldehyde was added to cells and cross-linked in formaldehyde at a final concentration of 1%, and then lysed by ChIP buffer. Chromatin DNA was sheared into 500-1000 bp fragments by ultrasonication. Chromatin was immunoprecipitated with the corresponding primary antibody after precleared, and then DNA was purified by DNA gel recovery kit (cat. WLA052a, Wanleibio, Shenyang, China) after reverse cross-linking for PCR. The primer sequences for ChIP assay were as follows: *Pim-1*, forward, 5′- CCCCTCCACAACCCAAATC -3′; reverse, 5′- CCAACCAGAAGACGCCCTAT -3′.

### 2.10. Dual-Luciferase Reporter Assay

To detect the role of *CEBPB* on the *Pim-1* promoter, the luciferase vector (pGL3-Basic-*Pim-1*-promotor) containing the fragment of the *Pim-1* promoter region and the *CEBPB* overexpression vector (*CEBPB*-OE) was constructed. Differentiated glomerular podocytes were cotransfected with pGL3-Basic-*Pim-1*-promotor and either *CEBPB*-OE or negative control when cultured to approximately 70% confluence. 48 h after transfection, luciferase activity was measured by Luciferase Activity Detection Kit (cat. KGAF040, KeyGEN, Nanjing, China).

### 2.11. Statistical Analysis

Data were recorded as mean ± standard deviation and analyzed by one-way analysis of variance and student *t* test method. The in vitro experiments were performed in triplicate and repeated at least three times. The *P* value smaller than 0.05 was considered statistically significant.

## 3. Results

### 3.1. The Expression of CEBPB Was Increased in the Development of LN

**Figure 1:**
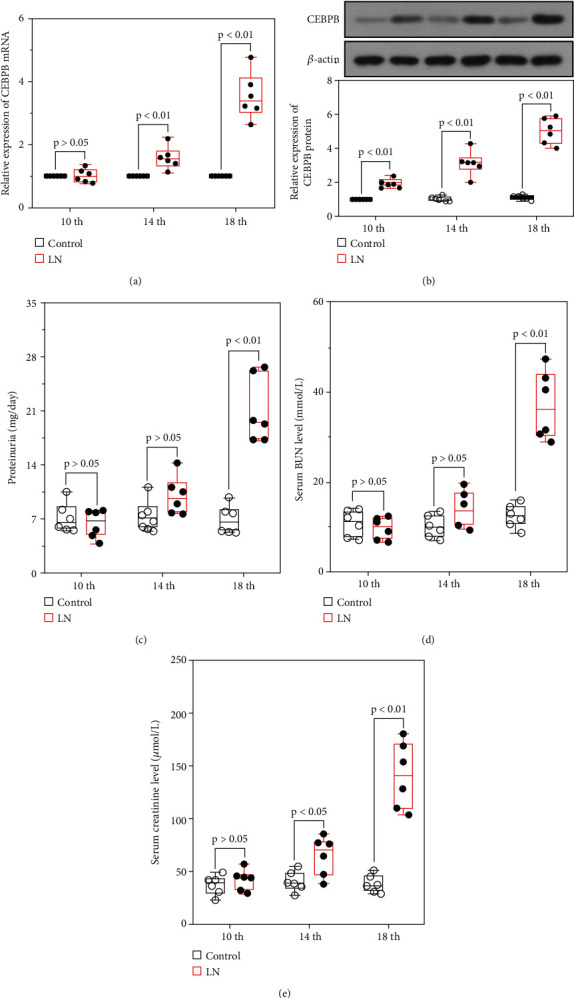
Expression of CEBPB was upregulated in the development of lupus nephritis in MRL/lpr mice. The mRNA expression (a) and protein levels (b) of CEBPB in kidney tissues of MRL/lpr or MRL/MPJ mice at 10th, 14th, and 18th week; 24-hour proteinuria level (c), serum BUN level (d), and serum creatinine level (e) in MRL/lpr or MRL/MPJ mice. (CEBPB, CCAAT/enhancer-binding protein *β*; BUN, blood urea nitrogen).

In the current study, MRL/lpr mice and MRL/MPJ mice were used to assess the expression of CEBPB with the development of LN. As shown in Figures 1(a) and (b), the mRNA and protein expression levels of CEBPB were elevated with increasing age in MRL/lpr mice compared with MRL/MPJ mice. Moreover, the levels of 24-hour proteinuria, serum BUN, and serum creatinine were increased in MRL/lpr mice (Figures 1(c)–1(e)). The results implied that the expression of CEBPB was increased in MRL/lpr mice with impaired renal function.

### 3.2. The Knockdown of *CEBPB* Alleviated the Pathological Features of LN in MRL/Lpr Mice

**Figure 2:**
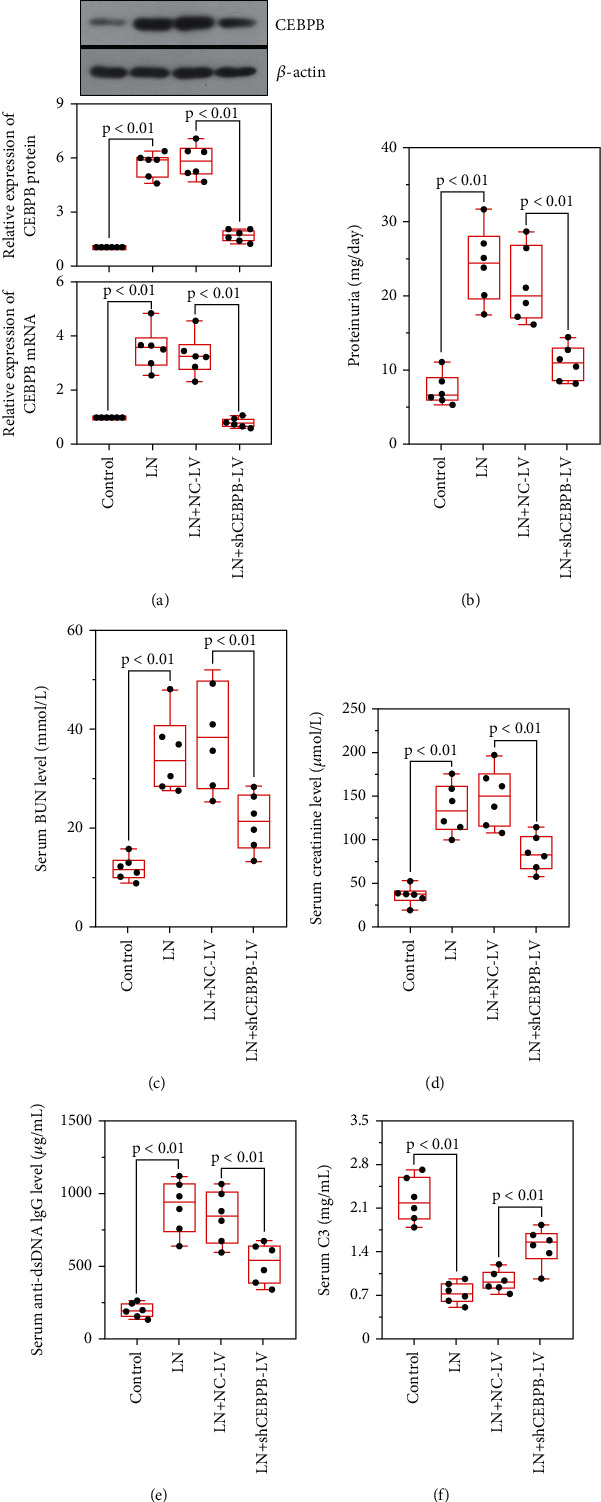
The knockdown of *CEBPB* alleviated the kidney damage in MRL/lpr mice. The mRNA and protein expression of CEBPB (a); 24-hour proteinuria level (b), serum BUN level (c), serum creatinine level (d), serum anti-dsDNA IgG level (e), and serum complement C3 level (f) in MRL/lpr mice injected with sh*CEBPB* lentivirus. (CEBPB, CCAAT/enhancer-binding protein *β*; sh*CEBPB*, *CEBPB*-short hairpin RNA; BUN, blood urea nitrogen).

**Figure 3:**
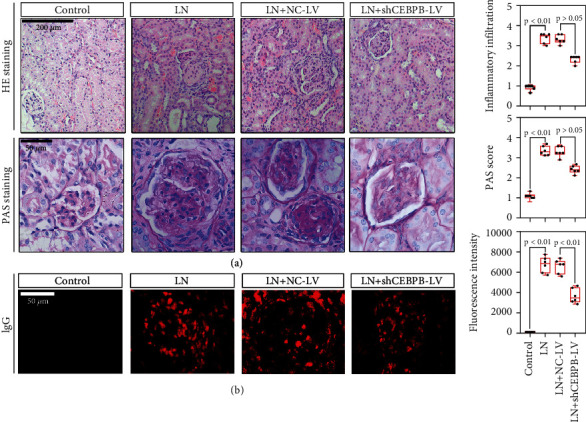
The knockdown of *CEBPB* alleviated the kidney damage on histopathology in MRL/lpr mice. Representative images of H&E (200 *μ*m) and PAS (50 *μ*m) staining (a) and immunofluorescence detection of IgG (50 *μ*m) (b) of kidney tissues in CEBPB-downregulated MRL/lpr mice. (CEBPB, CCAAT/enhancer-binding protein *β*; H&E, hematoxylin-eosin; PAS, Periodic Acid-Schiff).

Tail vein injection of sh*CEBPB* lentivirus into MRL/lpr mice downregulated the expression of CEBPB both in mRNA and protein levels in kidney tissues of LN mice (Figure 2(a)). Simultaneously, the typical characteristics of LN were ameliorated by downregulation of *CEBPB*, as evidenced by reduced 24-hour proteinuria, serum BUN, serum creatinine, anti-dsDNA IgG level, and elevated C3 level, that compared with the LN+NC-Lv mice (Figures 2(b)–2(f)). A similar changing pattern of CEBPB was observed in histological sections of kidney tissues. As shown in Figure 3(a), after H&E and PAS staining, the kidney tissue of MRL/lpr mice showed LN pathological damage, characterized by diffuse glomerulonephritis and PAS-positive material deposition, while there was a trend that this phenomenon was alleviated after sh*CEBPB* lentivirus treatment. In addition, less IgG deposition was detected in sh*CEBPB* lentivirus treated MRL/lpr mice by immunofluorescence (Figure 3(b)). These results show the effectiveness of targeting CEBPB in alleviating renal pathology symptoms of LN mice.

### 3.3. The Knockdown of *CEBPB* Inhibited the Activation of NLRP3 Inflammasomes in MRL/Lpr Mice

**Figure 4:**
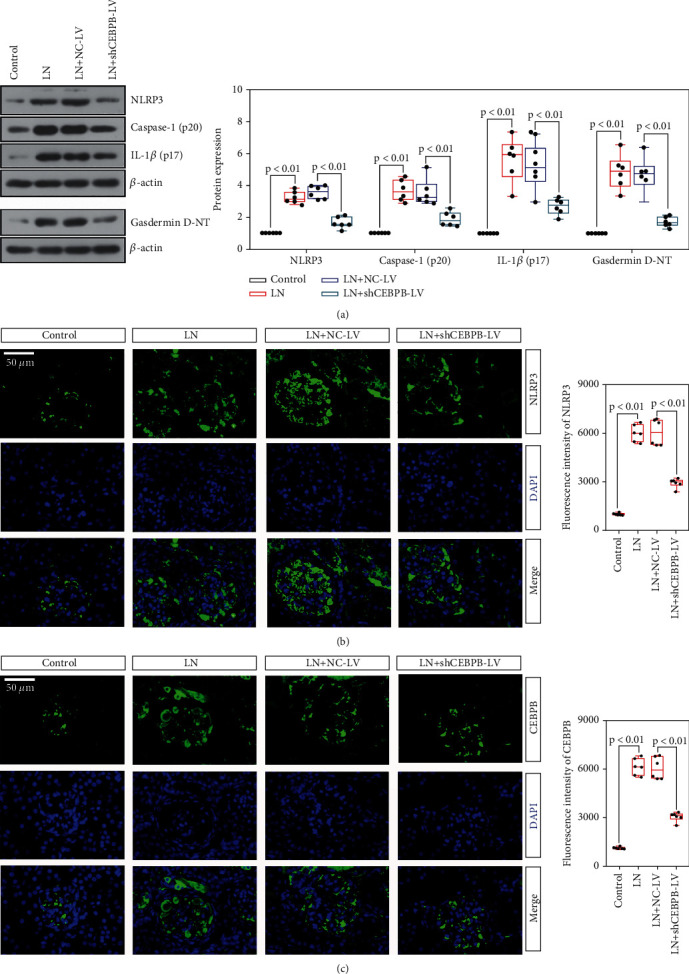
The knockdown of *CEBPB* inhibited the activation of NLRP3 inflammasomes in MRL/lpr mice. Relative expression of key proteins in the inflammasome-related signaling pathway in MRL/lpr mice after CEBPB downregulation (a); representative images of immunofluorescence detection of NLRP3 (b) and CEBPB (c) expression (50 *μ*m) in MRL/lpr mice with knockdown of *CEBPB*. (CEBPB, CCAAT/enhancer-binding protein *β*).

The activation of the inflammasome is a typical phenomenon associated with LN. Thus, we further assessed the effects of *CEBPB* knockdown on inflammasome activation in kidney. The expression of key proteins (NLRP3, caspase-1, and IL-1*β*) of the inflammasome signaling pathway and the hallmark protein of pyroptosis (Gasdermin D-NT) were evaluated in MRL/lpr mice. The results of western blot showed that the expression level of these proteins in sh*CEBPB* lentivirus treated mice was lower than that of MRL/lpr mice with negative control lentivirus (Figure 4(a)). In addition, the results of immunofluorescence showed that the expression of NLRP3 decreased along with the decrease of CEBPB level in the MRL/lpr mice with sh*CEBPB* lentivirus treatment (Figures 4(b) and 4(c)). Thus, we concluded that CEBPB may be involved in the mechanism contributing to NLRP3 inflammasome activation.

### 3.4. The Knockdown of *CEBPB* Inhibited the Activation of NLRP3 Inflammasomes in Glomerular Podocytes

**Figure 5:**
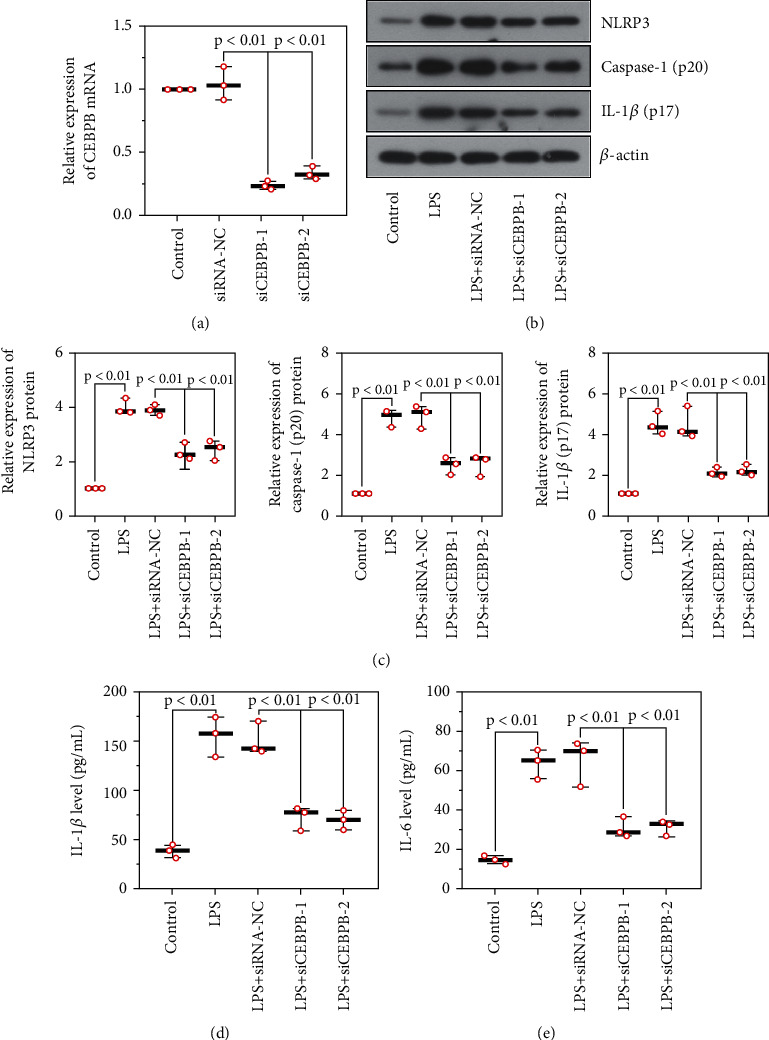
The knockdown of *CEBPB* inhibited the activation of NLRP3 inflammasomes in glomerular podocytes. Interference efficiency of *CEBPB* siRNAs (a). Protein expression of inflammasome-related proteins in podocytes stimulated with LPS and ATP (b, c). The content of IL-1*β* (d) and IL-6 (e) in the cell supernatant. (CEBPB, CCAAT/enhancer-binding protein *β*; ATP, adenosine triphosphate; LPS, lipopolysaccharides).

To obtain glomerular podocytes with low expression of CEBPB, two interfering fragments (si*CEBPB*1 and si*CEBPB*2) were transfected into differentiated glomerular podocytes using high-efficiency transfection reagents. The result showed that the mRNA expression of *CEBPB* was reduced in si*CEBPB* transfected glomerular podocytes (Figure 5(a)) NLRP3 expression was reduced after treatment with si*CEBPB* compared to vehicle-treated glomerular podocytes, and the expression of caspase-1 p20 and IL-1*β* p17 were also decreased (Figures 5(b) and 5(c)). Similarly, the knockdown of *CEBPB* reduced the content of IL-1*β* and IL-6 in the cell supernatant of LPS treated glomerular podocytes (Figures 5(d) and 5(e)). The above results indicated the involvement of CEBPB in the NLRP3 inflammasome activation process was associated with LN.

### 3.5. The Effect of CEBPB on *Pim-1* Promoter

**Figure 6:**
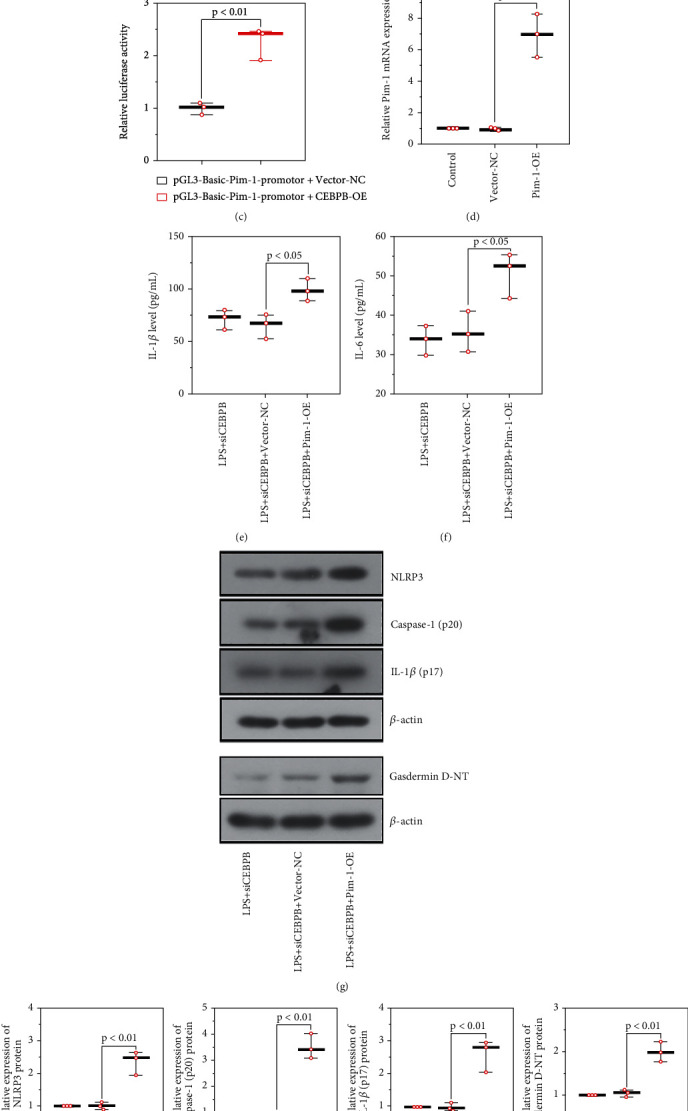
CEBPB regulated the activation of NLRP3 inflammasomes in glomerular podocytes via the regulation of Pim-1. The mRNA expression level of *Pim-1* in *CEBPB* knockdown podocytes (a); the binding of *Pim-1* promoter and CEBPB detected by chromatin immunoprecipitation (b) and dual-luciferase reporter assay (c); the transfect efficiency of *Pim-1* overexpression (d); the content of IL-1*β* (e) and IL-6 (f) in the cell supernatant; Protein expression of key proteins in the inflammasome-related signaling pathway (g, h). (CEBPB, CCAAT/enhancer-binding protein *β*).

As described in Figure 6(a), the mRNA expression of *Pim-1* was reduced in glomerular podocytes transfected with si*CEBPB*-1/2. Chromatin immunoprecipitation was used to detect the binding of *Pim-1* promoter and CEBPB. In Figure 6(b), the DNA fragment binding to CEBPB antibody and the input (positive control) were shown on the same size band, suggesting CEBPB can bind to the *Pim-1* promoter. Dual-luciferase reporter assay was used to analyze the effect of CEBPB on *Pim-1*. As shown in Figure 6(c), the luciferase activity was enhanced in CEBPB-overexpressed cells, indicating that CEBPB can promote the transcriptional activity of *Pim-1* activity.

### 3.6. CEBPB Regulated the Activation of the NLRP3 Inflammasome in Glomerular Podocytes via the Regulation of Pim-1

*Pim-1* overexpression vector (*Pim-1*-OE) was constructed to further investigate the regulatory effect of *Pim-1* on glomerular podocytes. As shown in Figure 6(d), the mRNA expression of *Pim-1* was greatly increased after *Pim-1* overexpression in glomerular podocytes. Further, the knockdown of *CEBPB*-induced reduction of IL-1*β* and IL-6 levels in LPS-treated glomerular podocytes was reversed by overexpression of *Pim-1* (Figures 6(e) and 6(f)). Similarly, CEBPB downregulation-induced suppression of inflammasome and pyrolysis were also abrogated by the upregulation of *Pim-1*, these were demonstrated by the increased NLRP3, IL-1*β* p17, caspase-1 p20, and Gasdermin D-NT levels (Figures 6(g) and 6(h)). The result illustrated that the inhibition of *CEBPB* downregulated the expression of *Pim-1*, and the overexpression of *Pim-1* reversed the inhibition of NLRP3 inflammasome induced by *CEBPB* deficiency in glomerular podocytes.

## 4. Discussion

Although considerable progress in treatment methods, LN remains a huge challenge for clinical treatment because patients often suffer from severe adverse effects and drug resistance, highlighting the need to discover new and effective therapeutic targets. In the present study, we evaluated the effect of *CEBPB* knockdown on LN symptoms in MRL/lpr mice and investigated its possible mechanisms. Results showed that knockdown of *CEBPB* attenuated LN in MRL/lpr mice, which was accompanied by the reversal of kidney pathological damage. Mechanistically, this effect may be achieved by the suppression of NLRP3-related inflammasomes. We also paid attention to the regulation of *CEBPB* on *Pim-1* in the development of LN in glomerular podocytes and found that the knockdown of *CEBPB* could downregulated the expression of *Pim-1* to improve LN and suppress the activation of the inflammasome. For this reason, our research indicated that CEBPB may be a promising target for the treatment of LN.

As a transcription factor, CEBPB is widely expressed in numerous cells and plays critical roles in the process of senescence, differentiation, proliferation, and inflammation [11–13]. Previous studies have determined a key role of CEBPB in SLE pathogenesis, which expression was increased in SLE patients [14]. In the current study, we found that CEBPB was upregulated with the increasing age in kidney tissues of MRL/lpr mice. Interestingly, the relative expression of mRNA did not correspond well with the protein expression in 10th mice. We speculated that the possible reason were as follows. Firstly, there are several processes occurred after transcription including posttranscriptional processing, degradation of transcripts, translation, and posttranslational modification [15]. The differences of the gene transcription and translation may be resulted from these processes or the regulation of other transcription factors. Then, there is a time interval between the gene transcription and translation, and the mRNA may be degraded when the protein expression levels are increased. Moreover, herein, a novel and important finding is that CEBPB expression is positively correlated with LN disease activity. As we all know, LN is one of the most serious forms of organ damage in SLE patients, and the resulting proteinuria induces renal injury. An abnormal increase in the serum BUN and creatinine levels also represents the occurrence of renal injury [16]. The positive correlation between CEBPB expression level and the anti-dsDNA antibody and the negative correlation with C3, suggesting that CEBPB is associated with LN activity. Moreover, complement deficiency impairs regulation and removal of autoantigens, whereas complement factors directly cause renal inflammation and immunopathology related to immune complex production [17]. In LN, nephritogenic autoantibodies are produced by B cells with the help of T cells [18]. The results of this study indicate that the treatment of sh*CEBPB* lentivirus could improve renal function and pathological damage via reducing the CEBPB expression in the kidney.

The published data have demonstrated that CEBPB is a key transcription factor to modulate inflammatory cytokine expression and is abnormally expressed both *in vitro* and *in vivo* [19]. Recent research reported that CEBPB downregulation could decrease inflammation, endoplasmic reticulum stress, apoptosis, and promote autophagy [20]. NLRP3 inflammasome is a molecular complex that activates caspase-1 and leads to the production of inflammatory cytokines such as IL-1*β* [21]. According to a new finding, activation of the NLRP3 inflammasome led to proteinuria and podocyte injury in lupus nephritis [22]. These findings are consistent with our research and indicate that the NLRP3 inflammasome is activated in podocytes by CEBPB and contributes to the pathogenesis of LN. Recently, the role of NLRP3 inflammasome in pyroptosis has received more and more attention [23–25]. Pyroptosis is an inflammatory form of programmed cell death and the Gasdermin D is the mediator of pyroptosis [26]. Recent key studies have identified that NLRP3-mediated pyroptosis was activated by CEBPB, which contributed to the promotion of acute kidney injury [27]. Further investigation in the present study revealed that the CEBPB inhibition improved the abnormal expression of NLRP3 and pyroptosis both *in vivo* and *in vitro*.

Another valuable and interesting finding in this study is that CEBPB promotes LN via Pim-1. Although as potent oncogenes, the key role of Pim kinases in immune regulation and inflammation has been widely investigated in recent years [28, 29]. Pim kinase is involved in immune and inflammation regulation through multiple mechanisms, including activating NK cells to induce the production of cytokines (e.g. IL-6) and activating the NF-*κ*B, mTOR, and MYC pathways [30]. In acute colitis, Pim-1 kinase inhibitors can reduce mucosal inflammation injury both *in vivo* and *in vitro* [31]. Recent research reported that Pim-1 is a critical regulator in LN pathogenesis of patients with SLE, and it modulates the activation of NLRP3 inflammasome via intracellular Ca^2+^ [9]. IL-1*β* production from human monocytes and the NLRP3 inflammasome activation were induced by immune complexes [32]. Caspase-1 is the central component of the NLRP3 inflammasome, and the activation of caspase-1 would cause pyroptosis by promoting the proinflammatory IL-1 cytokines family into their bioactive form (IL-1*β*) [33]. However, there is no report on the effect of CEBPB on Pim-1 during pyroptosis and NLRP3 inflammasome activation. Combined with our findings on *CEBPB*, the question of whether CEBPB would effectively regulate Pim-1 in LN was raised. In this study, we found that CEBPB was upregulated in the LN model, and CEBPB directly targeted the promoter region of *Pim-1* to promote its expression. Furthermore, knockdown of *CEBPB* could inhibit the release of inflammatory cytokines and pyroptosis related to NLRP3 inflammasome activation by downregulating Pim-1 in glomerular podocytes cells.

## 5. Conclusion

In summary, we identified the CEBPB-Pim-1 signal axis could promote the activation of NLRP3 inflammasome and pyroptosis of glomerular podocytes, which may serve as a therapeutic target against lupus nephritis.

## Data Availability

The data of this work is available on request to the corresponding author.
